# Comparative Analyses of the Impact of Different Criteria for Sepsis Diagnosis on Outcome in Patients with Spontaneous Subarachnoid Hemorrhage

**DOI:** 10.3390/jcm11133873

**Published:** 2022-07-04

**Authors:** Franz-Simon Centner, Mariella Eliana Oster, Franz-Joseph Dally, Johannes Sauter-Servaes, Tanja Pelzer, Jochen Johannes Schoettler, Bianka Hahn, Anna-Meagan Fairley, Amr Abdulazim, Katharina Antonia Margarete Hackenberg, Christoph Groden, Nima Etminan, Joerg Krebs, Manfred Thiel, Holger Wenz, Máté Elod Maros

**Affiliations:** 1 Department of Anesthesiology and Surgical Intensive Care Medicine, Medical Faculty Mannheim, University Medical Center Mannheim, University of Heidelberg, Theodor-Kutzer-Ufer 1-3, 68167 Mannheim, Germany; mariella.oster@medma.uni-heidelberg.de (M.E.O.); franz.dally@umm.de (F.-J.D.); johannes.sauter-servaes@umm.de (J.S.-S.); tanja.pelzer@umm.de (T.P.); jochen.schoettler@umm.de (J.J.S.); bianka.hahn@medma.uni-heidelberg.de (B.H.); anna-meagan.fairley@medma.uni-heidelberg.de (A.-M.F.); joerg.krebs@umm.de (J.K.); manfred.thiel@umm.de (M.T.); 2 Department of Orthopedics and Trauma Surgery, Medical Faculty Mannheim, University Medical Center Mannheim, University of Heidelberg, Theodor-Kutzer-Ufer 1-3, 68167 Mannheim, Germany; 3 Department of Neurosurgery, Medical Faculty Mannheim, University Medical Center Mannheim, University of Heidelberg, Theodor-Kutzer-Ufer 1-3, 68167 Mannheim, Germany; amr.abdulazim@umm.de (A.A.); katharina.hackenberg@umm.de (K.A.M.H.); nima.etminan@umm.de (N.E.); 4 Department of Neuroradiology, Medical Faculty Mannheim, University Medical Center Mannheim, University of Heidelberg, Theodor-Kutzer-Ufer 1-3, 68167 Mannheim, Germany; christoph.groden@umm.de (C.G.); holger.wenz@umm.de (H.W.); maros@uni-heidelberg.de (M.E.M.); 5 Department of Biomedical Informatics at the Center for Preventive Medicine and Digital Health (CPD-BW), Medical Faculty Mannheim, University of Heidelberg, Theodor-Kutzer-Ufer 1-3, 68167 Mannheim, Germany

**Keywords:** sepsis, sepsis criteria, infection, subarachnoid hemorrhage, sequential organ failure assessment score, systemic inflammatory response syndrome

## Abstract

Data on sepsis in patients with a subarachnoid hemorrhage (SAH) are scarce. We assessed the impact of different sepsis criteria on the outcome in an SAH cohort. Adult patients admitted to our ICU with a spontaneous SAH between 11/2014 and 11/2018 were retrospectively included. In patients developing an infection, different criteria for sepsis diagnosis (Sepsis-1, Sepsis-3_original, Sepsis-3_modified accounting for SAH-specific therapy, alternative sepsis criteria compiled of consensus conferences) were applied and their impact on functional outcome using the modified Rankin Scale (mRS) on hospital discharge and in-hospital mortality was evaluated. Of 270 SAH patients, 129 (48%) developed an infection. Depending on the underlying criteria, the incidence of sepsis and septic shock ranged between 21–46% and 9–39%. In multivariate logistic regression, the Sepsis-1 criteria were not associated with the outcome. The Sepsis-3 criteria were not associated with the functional outcome, but in shock with mortality. Alternative sepsis criteria were associated with mortality for sepsis and in shock with mortality and the functional outcome. While Sepsis-1 criteria were irrelevant for the outcome in SAH patients, septic shock, according to the Sepsis-3 criteria, adversely impacted survival. This impact was higher for the modified Sepsis-3 criteria, accounting for SAH-specific treatment. Modified Sepsis-3 and alternative sepsis criteria diagnosed septic conditions of a higher relevance for outcomes in patients with an SAH.

## 1. Introduction

More than 30% of patients with a spontaneous subarachnoid hemorrhage (SAH) develop infections during their hospitalization [1,2,3], and sepsis was identified as an independent risk factor for the impaired functional outcome [1,4,5,6]. Nevertheless, data on infectious complications and sepsis in patients with an SAH remain scarce, resulting in the need for further investigations [1,7].

Furthermore, an accurate diagnosis of sepsis remains challenging since a gold standard diagnostic test is not available [8,9,10,11,12,13,14]. Therefore, clinical sepsis criteria were formulated to identify sepsis [8,15,16]. In the Third International Consensus Definitions for Sepsis and Septic Shock (Sepsis-3), the previously used criteria based on systemic inflammatory response syndrome (SIRS) [15] were replaced by criteria that use the Sequential Organ Failure Assessment (SOFA) score [8,17]. Simultaneously, Sepsis-3 authors encouraged the refinement of the clinical sepsis criteria in different patient populations and clinical settings, as well as the development of enhanced sepsis criteria as necessary research objectives [17,18,19].

When studying sepsis criteria, two factors were identified as systematic weaknesses: first, the heterogeneity in the underlying illnesses of patients [20,21], and second, the presence of septic signs already on admission in general ICU populations, with the sepsis onset preceding the observation window of the ICU electronic health records (EHR) [17,22]. These obstacles were absent in the here analyzed SAH cohort. Patients with an SAH suffer from a non-infectious cerebral insult with a distinct onset. Included patients were admitted directly to the ICU upon hospital arrival after an SAH ictus and stayed for at least 12 consecutive days, owing to the risk of developing delayed cerebral ischemia (DCI) [23]. This provided comprehensive data to diagnose infectious events and investigate different established approaches to sepsis diagnosis. In addition, a compiled subset of diagnostic criteria for sepsis-associated organ dysfunction proposed in international consensus conferences [16,24] was used to allow for the diagnosis of sepsis and septic shock by alternative criteria.

The objective of this study was to

1. contribute data on infectious complications in SAH patients,
2. compare frequencies of sepsis and septic shock, respectively, according to different criteria, and
3. evaluate the impact of different sepsis detection methods on the functional outcome and mortality.

This approach was chosen to improve the diagnosis of sepsis in patients suffering from an SAH and contribute to an improved understanding of the impact of different sepsis criteria on sepsis diagnosis in a model population, enabling the generation of new hypotheses for sepsis detection in future studies. 

## 2. Materials and Methods

### 2.1. Design and Setting

This single-center retrospective cohort study was conducted at the 25-bed ICU of the Department of Anesthesiology and Surgical Intensive Care Medicine at the University Medical Center Mannheim, Germany. The Medical Ethics Commission II of Medical Faculty Mannheim, University of Heidelberg, approved the study design as well as reanalysis of neuroradiological reports and imaging data (file numbers: 2019-1096R, 2017-825R-MA and 2017-828R-MA) and confirmed that patient consent was not required because of the retrospective nature of the study.

**Figure 1:**
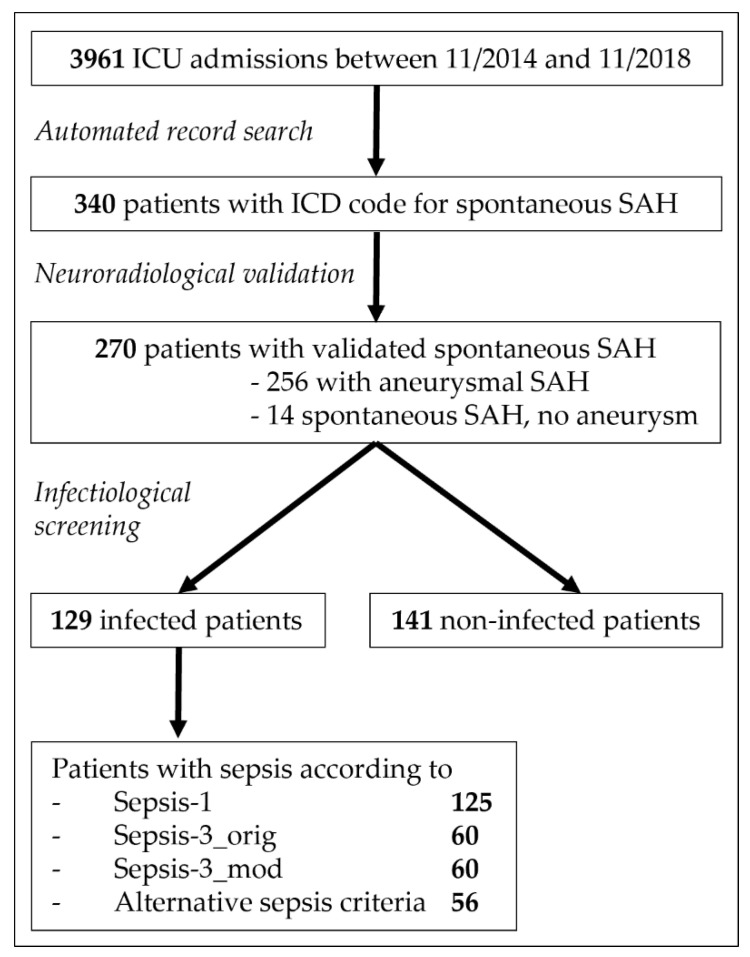
Flow chart of study cohort selection. Intensive Care Unit (ICU), International Classification of Diseases (ICD), subarachnoid hemorrhage (SAH).

After cross-checks for valid admissions with the hospital information system, data on all encounters (age ≥ 18 years) with complete ICU stay between 11/2014 and 11/2018 were extracted from the EHR database. Of these 3961 patients, 340 (8.6%) patients were preselected via ICD codes for spontaneous subarachnoid hemorrhage (code I60.-). After verification of spontaneous subarachnoid hemorrhage on initial computed tomography (CT) scan by an experienced neuroradiologist (H.W.), 270 patients were finally included as study cohort (Figure 1, for details on exclusion criteria see Table S1).

### 2.2. Clinical Assessment

All study patients were screened for healthcare-associated infections according to center for disease control (CDC) criteria [25] and the time of clinical manifestation was documented. For all patients developing infection, SIRS criteria were extracted by computational query, as described previously [26], and SOFA scores [27] were determined for 24-h periods by intensive care physicians. The cardiovascular SOFA component was scored in two separate ways:

1. according to the original definition by Vincent et al. [27], in the following referred to as “Sepsis-3_orig” criteria and
2. norepinephrine use was only counted for SOFA points if used to treat a dysfunction of the cardiovascular system and not if used for induced hypertension as part of DCI treatment [28]. The SOFA score resulting from this modified approach was used for criteria below called “Sepsis-3_mod”.

To ensure comparability of sepsis criteria application with original Sepsis-3 validation study [17], Sepsis-1 and -3 criteria were applied using an automated algorithm, as previously described in detail [26]. In infected patients, we not only applied the established sepsis criteria (Sepsis-1 and Sepsis-3) to diagnose sepsis and respective septic shock, but additionally compiled alternative sepsis criteria that were selected from diagnostic criteria for sepsis-associated organ dysfunction proposed in the international sepsis definitions of 2001 [13] and the surviving sepsis campaign of 2012 [24] (Table 1). Altogether, this resulted in eight distinct septic conditions (four different implementations for sepsis and four for septic shock, Table 2) being evaluated. 

Baseline characteristics (age, sex, length of ICU stay, and in-hospital mortality) were extracted from the EHR. Radiological imaging findings were evaluated by an experienced neuroradiologist (H.W.) including the modified Fisher scale. Clinical parameters were extracted and verified by intensive care physicians: pre-existing illnesses, World Federation of Neurological Surgeons SAH grading scale (WFNS) at initial presentation, type of aneurysm repair, occurrence of vasospasm and DCI, and modified Rankin Scale (mRS) at hospital discharge. To ensure comparability to similar studies [1,2,5], DCI was defined as the development of new focal neurological signs, deterioration in level of consciousness, or both, and/or the appearance of new infarction on CT or magnetic resonance imaging when the cause was most likely ischemia attributable to vasospasm after other possible causes of worsening had been excluded [31]. Angiographic vasospasm was considered an arterial narrowing on cerebral digital subtraction angiography (DSA) not attributable to atherosclerosis, catheter-induced spasm, or vessel hypoplasia, as determined by the DSA performing neuroradiologist [31]. Hydrocephalus was defined as neuroimaging signs of cerebrospinal fluid (CSF) circulation disorder and the need for CSF-drainage [1]. Rebleeding was defined as a new or progressive brain hemorrhage on CT before aneurysm repair [32,33]. 

### 2.3. Clinical Management

Patients with infections and sepsis were treated according to current recommendations [24,34]. Central venous access, PiCCO catheter (Pulsion Medical Systems, Munich, Germany), and Foley catheter placement were routinely performed in all study patients.

A cerebral CT scan including CT-angiography was initiated immediately after admission. Patients who were unable to protect their airway due to a decreased level of consciousness were intubated. Unconscious patients with space-consuming subdural hematoma or intracerebral hemorrhage and/or acute hydrocephalus or intraventricular hemorrhage were immediately treated surgically, i.e., hematoma removal, aneurysm repair, and, in selected cases, decompressive craniectomy. External ventricular drains (EVD) were placed via the Kocher point and in a tunneled fashion. In case of EVD placement (=205), patients received prophylactic antibiotics as single intravenous dose. Before aneurysm repair, systolic blood pressure was kept <140 mmHg. Decision on the modality of aneurysm repair was made in interdisciplinary consensus. Within the first 6–24 h after aneurysm repair, systolic blood pressures were maintained between 120 and 180 mmHg. After 24 h or following the exclusion of rehemorrhage after aneurysm repair, spontaneous systolic blood pressures were permitted, but generally maintained above 130 mmHg. Patients routinely received nimodipine 6 × 60 mg orally for at least 15 days following aneurysm repair.

From January 2016 an updated protocol for standardized detection and management of DCI was implemented in line with our previous studies [35,36], including thorough neurological examinations every 2–4 h in combination with a CT-perfusion screening protocol, especially for intubated and sedated patients, who were thereby not neurologically assessable: CT-perfusion measurements were performed on admission, 6–12 h after aneurysm repair, on day 3 or 4, as well as day 9 to 11 after SAH ictus. Additionally, DSAs were performed on admission and on day 6 to 9 after SAH ictus. Patients who developed clinical features of DCI underwent CT, CT-angiography, and CT-perfusion. If mean transit time in CT-perfusion was >1.5 times baseline, DSA was indicated. In case of persistent clinical or radiological features of DCI, the patients were treated according to a standardized, escalating treatment protocol: 1. induced hypertension with a targeted systolic blood pressure of >180 mmHg; 2. solitary intra-arterial nimodipine bolus applications during DSA; and 3. angiographic application of an intra-arterial catheter for continuous nimodipine administration over 48 h with CT-perfusion imaging in between each escalating step.

Before January 2016, management decisions were usually taken on an individual basis and patients were predominately treated according to the presence of angiographic vasospasm. Patients were neurologically assessed in ICU and underwent DSA on days 6 to 9 after aneurysm repair or immediately upon clinical deterioration for assessment of the presence of angiographic vasospasm. Induced hypertension was not applied as consistently. Patients with severe vasospasm and/or clinically relevant vasospasm or patients with progressive vasospasm on repeated DSA received an intra-arterial catheter for continuous nimodipine administration as first-line treatment. Angiographic vasospasm was monitored by a follow-up DSA after 72 h.

Patients with spontaneous SAH, in the absence of an aneurysm as the source of hemorrhage, received the same routine treatment described above, with bed rest for one week after SAH ictus followed by control DSA. In patients with perimesencephalic SAH, control DSAs were not routinely performed.

### 2.4. Statistical Analysis

All analyses were conducted using SAS software, version 9.4 (SAS Institute, Cary, NC, USA). Normally distributed variables were described using their means and standard deviations (SD), non-normally distributed variables with their medians, min–max ranges, lower and upper quartile (LQ-UQ), and interquartile ranges (IQR), while proportions were shown for categorical variables. The Cohen’s kappa statistic was used to assess agreement between septic populations according to different sepsis criteria [37]. Primary outcome of interest was functional outcome measured by mRS at hospital discharge. Due to class imbalances, mRS values were dichotomized at ≤3 vs. >3 (cut-off chosen to be comparable [1,2]). As, to our knowledge, Gonçalves et al. were the only group investigating Sepsis-3 criteria in SAH patients using mRS as outcome [1], we chose a similar modeling approach using stepwise logistic regression automated variable selection [38,39]. However, as modification to Gonçalves et al., we used the default settings of the stepwise selection algorithms that requests stepwise selection based on the Schwarz Bayesian information criterion (SBC) as defined in PROC LOGISTIC. Explanatory variables that achieved a significance level of <0.1 were allowed into the model (SLENTRY = 0.1) and a significance level of <0.2 was required for a variable to remain in the model (SLSTAY = 0.2). Intermediate models were compared using the likelihood-ratio test [40]. We reconstructed the final model regarding functional outcome (mRS ≤ 3 vs. >3) at discharge by Gonçalves et al., which included age, sex, WFNS 4-5, hydrocephalus, DCI, pneumonia, and Sepsis-3. We refitted this model by replacing Sepsis-3 with one of eight sepsis implementations (Table 2), respectively. Further, we introduced the factor variable “updated DCI protocol” to adjust for the above-described changes in DCI management (Npre = 69, 26%; Npost = 201, 74%). For variables that were significantly associated with the primary outcome (mRS) in logistic regression analysis, we used Fisher’s exact test as independence test on the contingency table of categorical variables and investigated multicollinearity using Spearman’s rank correlation [41] and variance inflation factor (VIF) analysis [42]. As secondary outcome, we followed recommendations regarding evaluation of sepsis criteria [9,10] and modeled death as binary outcome variable, which occurred in 55 patients. 

Our main explanatory variables of interest in all primary and secondary outcome models were Sepsis-1, Sepsis-3_orig, Sepsis-3_mod, and sepsis, according to alternative sepsis criteria (Table 1), as well as the consecutive implementations of septic shock to compare the impact of these criteria. As our analyses were explorative and served to generate hypotheses, *p*-values were not adjusted for multiple testing. *p*-values < 0.05 were considered significant. Additionally, we performed sensitivity analyses (i.e., all primary and secondary models were repeated) in order to assess the comparability of the automated variable selection approach across different patient populations and to investigate the accrual effect of patient selection made by Gonçalves et al. in their cohort, as they excluded patients who died within 48 h after ICU admission [1].

## 3. Results

Of the 270 enrolled patients with spontaneous SAH, 256 (95%) had an aneurysmal SAH, and in 14 patients (5%), no aneurysm was detected. The baseline characteristics of the study cohort are shown in Table 3. A total of 153 patients (57%) had a poor functional outcome at hospital discharge (mRS > 3) and in-hospital mortality was 20% (55 patients).

### 3.1. Healthcare-Associated Infections in SAH Patients

Of the 270 SAH patients, 129 (48%) developed at least one infectious event. In total, 197 infectious events occurred (Table 4). Seventy-eight (29%) patients had one distinct episode of infection, thirty-six (13%) patients had two, thirteen (5%) had three, and two (1%) patients had four. The median time between admission and the first diagnosis of infection was 6 days (min–max 0–29, LQ–UQ: 4–10, IQR: 6). Of these 197 infectious events, 13 (7%) led to a secondary bloodstream infection (thereof, five urinary tract infections (UTI), four pneumonias, three meningitides, and one skin infection). Of 104 events with a detection of ≥105 colony forming units (CFU)/mL in urine, 73 (70%) fulfilled the CDC criteria for UTI, while 31 (30%) events constituted bacteriuria. Of 49 pneumonias, 17 (35%) were ventilator-associated. Of 205 EVDs, 178 (87%) were not associated with meningitis, but all 27 meningitis cases had an EVD. Of fourteen patients with a non-aneurysmal spontaneous SAH, four (29%) developed an infection.

### 3.2. Sepsis According to Established Sepsis Criteria

According to Sepsis-1 criteria, 125 of 129 patients with an infection were classified as septic (97% of infected patients and 46% of the total cohort, respectively, Table 5). When using Sepsis-3 criteria, 60 patients were detected as septic by both SOFA scoring approaches (Sepsis-3_orig and Sepsis-3_mod), resulting in a sepsis frequency of 47% in patients with an infection and 22% in relation to the total cohort (Table 5). However, these two approaches to the scoring of the cardiovascular SOFA component identified partially distinct populations: 49 patients (49/60, 82%) were identified by both Sepsis-3 implementations, while the other 18% of septic populations differed (kappa = 0.66, 95% confidence interval (CI) 0.53–0.79). This resulted in a mortality rate of 18% within the septic subpopulation for Sepsis-3_orig and of 22% for Sepsis-3_mod (Table 5).

### 3.3. Sepsis According to Alternative Sepsis Criteria

The incidence of sepsis according to alternative criteria to detect a sepsis-associated organ dysfunction (Table 1) was 21% (56/270) in the overall cohort and 43% (56/129) in the subpopulation of patients who developed an infection (Table 5). The median time until the first sepsis episode was 7 days after ICU admission (min–max 0–35; LQ-UQ: 4–11.5). The sepsis-causing infection and the associated pathogens are displayed in Table 4. Organ dysfunctions that were associated with sepsis according to alternative criteria and respective mortality are shown in Table S2. In SAH patients whose sepsis status was determined by alternative sepsis criteria, 25% of septic patients and 19% of non-septic patients died, respectively (Table 5). One of the 56 sepsis cases was observed in a patient with a non-aneurysmal SAH.

### 3.4. Agreement of Different Methods for Sepsis Detection

Pairwise agreement between alternative sepsis criteria and the other implementations of sepsis diagnosis was slight for Sepsis-1 criteria (kappa = 0.02, 95% CI −0.03–0.07), fair for Sepsis-3_orig (kappa = 0.34, 95% CI 0.18–0.50), and moderate for Sepsis-3_mod (kappa = 0.59, 95% CI 0.45–0.73) [37]. For different septic shock criteria, a slight agreement was observed between alternative sepsis criteria and Sepsis-1 (kappa = 0.10, 95% CI 0.03–0.16), while agreement with Sepsis-3_orig was moderate (kappa = 0.54, 95% CI 0.35–0.72) and with Sepsis-3_mod, substantial (kappa = 0.72, 95% CI 0.57–0.86).

### 3.5. Impact of Different Sepsis Criteria on Functional Outcome

**Figure 2:**
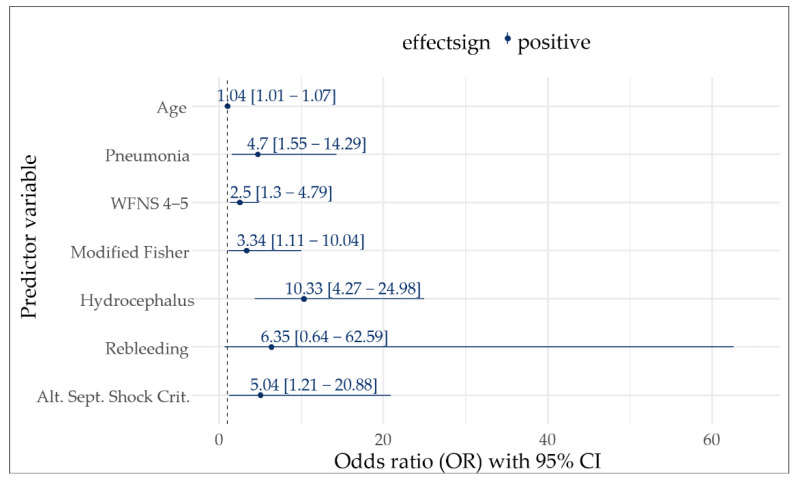
Multivariate logistic regression model-based odds ratios with 95% CI for functional outcome at discharge. Poor functional outcome was defined as modified Rankin Scale > 3. Displayed are only those variables that remained in the model during stepwise variable selection process.

In univariate analyses, age (*p* < 0.001), infection (*p* < 0.001), pneumonia (*p* < 0.001), WFNS (*p* < 0.001), a modified Fisher score (*p* < 0.001), hydrocephalus (*p* < 0.001), and DCI (*p* = 0.045) were associated with an unfavorable outcome (mRS > 3) at hospital discharge, as well as all applied definitions for sepsis and septic shock (Table S3). For multivariate analyses, we repeated all selection processes with each distinct sepsis definition separately. Alternative criteria for septic shock were the only implementation that remained independently associated with a poor functional outcome (odds ratio (OR) 5.0, 95% CI 1.2–20.9, *p* = 0.026, Figure 2) while Sepsis-3_mod septic shock stayed in the model, but slightly missed the threshold for significance (OR 3.2, 95% CI 0.9–11.4, *p* = 0.069; Table S3). Further variables that displayed independent associations with a poor functional outcome included age, pneumonia, WFNS, a modified Fisher score, and hydrocephalus (Figure 2). It is of note that Fisher’s exact test showed a significant positive association (co-occurrence) of pneumonia and alternative septic shock criteria (OR 11.59, 95% CI 4.4–32.1, *p* < 0.001). However, no relevant multicollinearity was detected for the outcome-associated variables. A detailed correlation matrix and VIFs of these variables are shown in Figure S1 and Table S4.

In sensitivity analyses, we excluded patients who died within 48 h after ICU admission to ensure comparability with Gonçalves et al. [1]. Of all eight investigated sepsis operationalizations, alternative criteria for septic shock remained the only one that was associated with a poor functional outcome in multivariate analysis (OR 4.4, 95% CI 1.0–19.1, *p* = 0.049). We could reproduce Gonçalves et al.’s findings, in that age, WFNS, hydrocephalus, and DCI were independently associated with an unfavorable outcome. Additionally, in our model, pneumonia showed a significant association with unfavorable outcomes (Table S5).

### 3.6. Impact of Different Sepsis Criteria on Mortality

**Figure 3:**
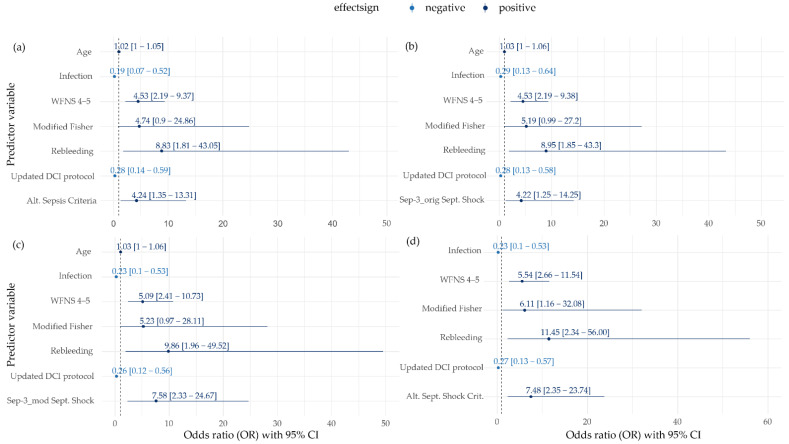
Multivariate logistic regression model-based odds ratios with 95% CI for in-hospital death at discharge. Different criteria for diagnosis of sepsis ((**a**) alternative sepsis criteria; (**b**) septic shock Sepsis-3_orig; (**c**) septic shock Sepsis-3_mod; (**d**) alternative septic shock criteria) were added one at a time to the pool of variables using a separate stepwise selection process to investigate the association with in-hospital mortality. Displayed are only those variables that remained in the model during variable selection.

In univariate analyses, age (*p* = 0.002), WFNS (*p* < 0.001), modified Fisher (*p* = 0.035), hydrocephalus (*p* = 0.005), rebleeding (*p* = 0.005), angiographic vasospasm (*p* = 0.038), updated DCI protocol (*p* = 0.007), and alternative criteria for septic shock (*p* = 0.038) were significantly associated with death (Tables S6 and S7). Multivariate logistic regression analyses revealed that sepsis according to alternative criteria was the only sepsis implementation that was independently associated with mortality (OR 4.2, 95% CI 1.4–13.3, *p* = 0.013, Figure 3a). Furthermore, WFNS and rebleeding remained independently associated with death, while an updated DCI protocol and the development of an infection were independently associated with survival (Figure 3a–d). Except for Sepsis-1 implementation, all applied criteria for septic shock were associated with death. The highest odds ratios were observed for alternative criteria (OR 7.5, 95% CI 2.4–23.7, *p* < 0.001) and Sepsis-3_mod (OR 7.6, 95% CI 2.3–24.7, *p* < 0.001) compared to Sepsis-3_orig (OR 4.2, 95% CI 1.3–14.3, *p* = 0.020) (Figure 3b–d). The independent impact of septic shock according to alternative criteria and Sepsis-3_mod on death was confirmed in sensitivity analyses, excluding SAH patients who died within 48 h after ICU admission. However, the criteria for sepsis, according to alternative sepsis criteria (*p* = 0.069), and for septic shock, according to Sepsis-3_orig (*p* = 0.095), both marginally missed the significance threshold for their association with mortality (Tables S8 and S9).

## 4. Discussion

In this study, we compared the impact of different criteria for sepsis diagnosis on sepsis frequencies and functional and survival outcomes in a cohort of 270 patients with an SAH. By choosing an SAH as the underlying condition of the study population, two important prerequisites for studies on sepsis criteria could be established [17,20,21,22]: homogeneity in the underlying disease within the study population and development of infectious complications under full surveillance.

We took this approach to improve the diagnosis of sepsis in patients with an SAH and contribute to an improved understanding of the impact of different sepsis criteria on sepsis diagnosis in a model population, enabling the generation of new hypotheses for sepsis detection in future studies. The present study adds several new insights to the topic. In each of the few previous studies on sepsis in patients with SAH, sepsis was diagnosed by solely one implementation of sepsis criteria [1,2,3,4,5,43]. With the present study, we were, to our knowledge, the first to simultaneously apply the previously separately used sepsis criteria to diagnose sepsis and septic shock within the same SAH cohort and compare the impact with respect to epidemiology and patients’ outcome. Furthermore, we presented a modified scoring method of the SOFA score for sepsis diagnosis by Sepsis-3 criteria, accounting for SAH-specific induced hypertension. Finally, we proposed alternative criteria to detect a sepsis-associated organ dysfunction as a need for enhancements of the Sepsis-3 criteria has been identified in general [17,18,19,26,44,45] and, specifically, in the context of SAHs [1].

### 4.1. Incidence of Healthcare-Associated Infections in SAH Patients

We used the CDC criteria to identify healthcare-associated infections [25], which resulted in a higher infection rate (48%) in our SAH cohort than reported in comparable studies (16–38%) [1,5,46,47,48,49]. Similar to previous reports [46,47], the most prevalent infections were UTIs, pneumonias, and meningitides. The occurrence of UTIs was 27%, and was comparable to that in a nationwide US cohort of 24% [50]. Additionally, the frequency of pneumonia was comparable between studies (16% in [1,5], 17% in [46], and 18% in our collective; Table 4). Meningitides were more frequently observed in our cohort (10%) compared to other collectives (5% in [2] and 6% in [1]), which could be caused by higher numbers of EVD placements (37% in [2] vs. 76% in our cohort). Previously reported numbers of meningitides in 11% of patients with EVD placement align with our observations [51]. The frequency of primary bloodstream infections was observed in 8% of patients in our cohort and by Frontera et al. [2], thereby ranging between further studies (1% in [1] and 16% in [5]).

While reports on infections and corresponding pathogens in SAH patients exist [46,47], we are not aware of another report on sepsis-causing infections and associated pathogens (Table 4). With methicillin-sensitive *Staphylococcus aureus* and *Haemophilus influenzae* being the most common pathogens identified in sepsis-causing pneumonias and Escherichia coli in sepsis-causing UTIs, there was no difference in the pathogen spectrum regarding pneumonias and UTIs in an SAH in general [46,47].

Pneumonia was by far the most frequent sepsis-causing infection. Two key findings concerning pneumonia in patients with an SAH have been shown before: one, a considerably increased incidence of pneumonia in patients with a higher WFNS grade, which was possibly attributable to SAH-associated immunodepression [52], and two, a significant correlation between pneumonia and a poor functional outcome three months after discharge even after adjusting for SAH grading [2]. The latter findings were confirmed in our cohort for the functional outcome at hospital discharge (OR ranging between 4.7 to 7.6 depending on the used sepsis definition) whereas Gonçalves et al. did not observe an independent association [1]. Although pneumonia showed significant co-occurrence with alternative septic shock criteria in our cohort, it stayed significantly associated with the functional outcome even after adjusting for alternative septic shock criteria and the further mRS-associated parameters (Figure 2).

### 4.2. Incidence of Sepsis in Patients with SAH

Quality data on sepsis in patients with SAH is limited [1,5,7]. Imprecise sepsis definitions were a concern in previous studies [1]. Although positive blood cultures occur in only one third of sepsis cases [53], it was used as a sepsis definition in some studies [2,4,54]. Consequently, a low sepsis frequency of 5% was found in a cohort of SAH patients using this definition [4]. In a nationwide analysis of infections in patients after SAH, sepsis frequency was also 5%, but ICD-codes were used for sepsis diagnosis [50] which has been identified to be imprecise for that purpose [44]. Other studies on sepsis in patients with an SAH used the SIRS-based Sepsis-1 criteria for sepsis diagnosis [3,43,55], reporting sepsis frequencies between 28% and 38%, compared to 46% in our study. The general criticism of SIRS as imprecise sepsis criteria [56,57] is arguably even more justified in patients with SAH, as SIRS was identified to be present in more than 85% of patients with SAHs across studies independent from infection due to SAH-associated systemic inflammation [55,58,59].

Gonçalves et al. and Bogossian et al. were the first to investigate the Sepsis-3 definition in patients with SAHs and observed sepsis frequencies of 28% and 22%, respectively [1,5]. These findings agree with our results, with sepsis frequencies of 22% according to the two applied implementations of Sepsis-3, and 21% when using alternative criteria. Furthermore, numbers for Sepsis-3 septic shock in SAH patients reported with 12% and 11%, respectively [1,5], were similar in our study with 9% and 10%, depending on implementation (Table 5).

### 4.3. Impact of Different Sepsis Criteria on Outcome in SAH Patients

Sepsis was shown to be independently associated with persistent new cognitive impairment and functional disability among survivors in general populations [60] and presumably contributes to brain dysfunction after an SAH as a second hit to a vulnerable brain [1]. Hence, an impact of sepsis on the functional outcome in patients with an SAH could be presumed [1]. Three previous studies found an independent association of sepsis with a poor functional outcome [1,2,6], while Bogossian et al. observed no significant correlation for sepsis, but did for septic shock (OR 6.4) [5]. The latter findings were confirmed in our cohort, in that septic shock, according to alternative criteria, was the only one of the implementations that displayed an independent association with the poor functional outcome (OR 5.0). These results remained robust in the sensitivity analyses. 

In addition to the poor functional outcome which is frequently used in studies on SAHs, we chose mortality as the secondary outcome since it was postulated as the outcome of choice for the assessment of the criterion validity of the sepsis criteria [9,10] and was used for the original evaluation of the Sepsis-3 criteria [17]. In previous studies on the causes of death in patients with an SAH, extracranial causes, including systemic infection, contributed to only 2% of the observed deaths in patients with a poor-grade SAH (WFNS 4–5) [61], while in a general SAH population, sepsis was found not to be independently associated with in-hospital mortality [54]. However, the robustness of these findings may be limited as imprecise sepsis definitions were used [54,61]. Sepsis-3 criteria were designed to capture a mortality above 10% for sepsis and 40% for septic shock in general populations [8,17,62]. Consequently, sepsis should have an independent effect on mortality, even in SAH patients, that is more pronounced than for septic shock [5]. However, in the only previous study investigating the Sepsis-3 criteria in patients with SAH for the outcome of mortality by Bogossian et al., neither sepsis nor septic shock displayed an independent association [5]. In our investigation, the alternative criteria for sepsis (OR 4.2) and all applied criteria for septic shock, except for Sepsis-1, were independently associated with death (Figure 3). The highest odds ratios were observed for septic shock according to modified Sepsis-3 and alternative criteria (OR 7.6 and 7.5). Results for these implementations remained robust during sensitivity analyses. In contrast, original Sepsis-3 septic shock did not remain independently associated with mortality in the sensitivity analysis, which could possibly explain the negative results by Bogossian et al. [5].

In summary, for septic shock, the Sepsis-3_mod and alternative criteria showed a superior and robust association with in-hospital mortality in patients with SAH compared to Sepsis-3_orig, and the alternative sepsis criteria additionally showed an association with the functional outcome. Thereby, septic shock according to alternative criteria was the only implementation that displayed the presumable impact of septic shock on the functional and survival outcome in SAH patients.

### 4.4. New Approaches to Sepsis Detection in SAH Patients 

Sepsis is currently defined as a life-threatening organ dysfunction caused by a dysregulated host response to infection [8]. SOFA-based sepsis criteria were introduced to detect this sepsis-defining organ dysfunction [8,17]. Simultaneously, Sepsis-3 authors encouraged the refinement of the sepsis criteria in different patient populations and clinical settings, as well as the development of enhanced sepsis criteria as necessary research objectives [17,18,19]. Recently, statistical modeling and machine learning-based algorithms demonstrated their potential for the evaluation and prediction of sepsis outcomes [6,13,14,48,63]. In this context, we here investigated two new approaches to sepsis detection: First, as in SAH patients, sepsis diagnosis can be confounded by the use of vasoactive drugs for induced hypertension in case of DCI [1,28]; in addition to using the original SOFA score, we modified the cardiovascular component to distinguish the use of norepinephrine for SAH-specific treatment from its use to treat a dysfunction of the cardiovascular system. Second, as in previous studies the fixed cutoff-values in the SOFA score were criticized [64,65] and the threshold of two SOFA points was found to be too low for specific sepsis detection in critically ill patients [26,66,67]; to address these concerns, we compiled alternative sepsis criteria that were selected from diagnostic criteria for sepsis-associated organ dysfunction proposed in international consensus conferences ([16,24], Table 1). 

The analyses based on these new approaches (Sepsis-3_mod and alternative sepsis criteria) showed a superior and robust association of septic shock with in-hospital mortality in patients with an SAH compared to Sepsis-3_orig septic shock. Moreover, the alternative sepsis criteria showed an association with death even in the case of sepsis and in the case of septic shock additionally with the functional outcome. We hypothesize that this superior relevance for the outcome of the alternative sepsis criteria might be due to the integration of additional features to detect sepsis-associated organ dysfunction and partly dynamic instead of fixed variable cutoffs compared to the SOFA score. Additionally, although not directly convertible into SOFA points in all categories, the alternative criteria in tendency require a higher severity of disease to turn positive than Sepsis-3 criteria. 

One approach to validate sepsis criteria, which was chosen to establish Sepsis-3 criteria [17], is to investigate their association with the adverse outcome [9,10,19]. Following this approach, as modified Sepsis-3 and alternative sepsis criteria showed a superior association with the adverse outcome in the here analyzed SAH cohort, they might be a promising approach to enhance the detection of septic conditions in SAH patients and, furthermore, in other populations, respectively. However, this needs to be tested in future prospective clinical studies in well-defined patient populations.

### 4.5. Limitations

Our study has several limitations. We present a single-center retrospective cohort study. Nevertheless, to our knowledge, this is the largest SAH cohort in which Sepsis-3 criteria have been investigated so far. To comprehensively describe infectious complications, the retrospective nature allowed for the integration of all available information independent from temporal occurrence. However, the retrospective design prevented conclusions on the cause–effect relations. Similar to other comparable studies [1,2,5], we did not adjust for the effect of the length of the ICU stay which captures potential bias to our results. However, results obtained in our study were shown to be robust by sensitivity analyses, omitting patients that died within the first 48 h upon ICU admission. The, here established, modified scoring of the cardiovascular SOFA component and the alternative criteria for sepsis diagnosis rely, to a certain extent, on clinical judgment, bearing the risk of subjectivity, and complicating automated operationalization. The updated DCI protocol in our institution displayed no impact on the functional outcome at discharge, but significantly impacted mortality, which is beyond the scope of this report, but should be the subject of future research. By adjusting for this protocol in multivariate analyses, we ensured that the presented results were robust.

## 5. Conclusions

Impact of sepsis on outcome in SAH patients was dependent on the applied criteria for its diagnosis. Therefore, our results emphasize the importance of accurate implementation and detailed reporting when using sepsis criteria. While the Sepsis-1 criteria were irrelevant for outcome, the association of the Sepsis-3 criteria with mortality was not observed for sepsis, but for septic shock. This association was stronger when a modification of the SOFA score accounting for SAH-specific treatment was applied. We compiled alternative criteria for the detection of a sepsis-related organ dysfunction that were independently associated with mortality for sepsis and septic shock, and additionally with the functional outcome in a shock state. Consequently, the modified Sepsis-3 and alternative sepsis criteria detected septic conditions of a higher relevance with respect to survival and the functional outcome in patients with SAH.

## Figures and Tables

**Table 1:** Alternative sepsis criteria to detect an organ dysfunction caused by a dysregulated host response to infection ^1^.

Organ System	Criteria for Organ Dysfunction
Cardiovascular	sepsis-induced hypotension (SBP < 90 mmHg or MAP < 70 mmHg) or new necessity of norepinephrine to reach MAP ≥ 70 mmHg or increase of norepinephrine ≥ 1.5 times baseline ^2^
Renal	urine-output < 0.5 mL/kg/h for ≥6 h ^3^ increase of serum creatinine ≥ 1.5 times baseline ^3^
Respiration	paO_2_/FiO_2_ < 250 in absence of pneumonia ^4^ paO_2_/FiO_2_ < 200 if pneumonia present ^4^
Liver	bilirubin > 2 mg/dL
Coagulation	thrombocytopenia (platelet count < 100,000/µL) or coagulation abnormalities (INR > 1.5)
CNS	septic encephalopathy (confusion or agitation) ^5^
Gastrointestinal	ileus or gut ischemia

Central Nervous System (CNS), Fraction of inspired Oxygen (FiO_2_), International Normalized Ratio (INR), Mean Arterial Pressure (MAP), partial pressure of arterial Oxygen (paO_2_), Systolic Blood Pressure (SBP); ^1^ modified from Surviving Sepsis Campaign 2012 ([24], their Table 2) and 2001 International Sepsis Definitions Conference ([16], their Table 1); ^2^ if norepinephrine was used previously to induce hypertension to treat DCI [28]; ^3^ thereby fulfilling at least kidney disease improving global outcomes (KDIGO) of acute kidney injury stage I [29]; ^4^ pneumonia according to CDC criteria [25]; ^5^ DCI or other acute SAH-associated alterations of the CNS had to be less likely than an association with infection. If both could be possible, confusion or agitation were not rated a septic encephalopathy.

**Table 2:** Criteria to diagnose sepsis and respective septic shock.

	Sepsis Criteria	Septic Shock Criteria
Sepsis-1	presence of ≥2 SIRS criteria within an infection period ^1^ [15]	sepsis according to Sepsis-1 + norepinephrine ≥ 0.1 µg/kg/min
Sepsis-3_orig	acute change in SOFA_orig ≥ 2 within an infection period ^1^ [8]	sepsis according to Sepsis-3_orig + norepinephrine ≥ 0.1 µg/kg/min + lactate > 2 mmol/L
Sepsis-3_mod	acute change in SOFA_mod ≥ 2 within an infection period ^1^	sepsis according to Sepsis-3_mod + norepinephrine ≥ 0.1 µg/kg/min + lactate > 2 mmol/L
Alternative sepsis criteria	presence of at least one organ dysfunction according to alternative sepsis criteria (Table 1) within an infection period ^1^	sepsis according to alternative sepsis criteria + dysfunction of cardiovascular system according to criteria (Table 1) + lactate > 2 mmol/L

Systemic Inflammatory Response Syndrome (SIRS), Sequential Organ Failure Assessment (SOFA); ^1^ To ensure comparability for evaluation of sepsis criteria, a time frame of 4 days around infection onset was implemented (including 2 days before the onset of infection, the day of infection onset itself, and 1 day after) as validated for robustness by Verboom et al. [30]). Sepsis was considered present if the respective criteria were fulfilled within this 4-day window around infection onset.

**Table 3:** Patient demographics and baseline characteristics of cohort with spontaneous SAH (N = 270).

Variables		N (%)
Age [y], median (LQ–UQ = IQR) (range)		57 (50–66 = 16) (18–91)
Female		182 (67)
Arterial hypertension		155 (57)
Smoking		94 (35)
Diabetes mellitus		23 (9)
Aneurysmal SAH		256 (95)
Spontaneous SAH without aneurysm identified		14 (5)
Thereof perimesencephalic SAH		5 (36)
WFNS grade	I	91 (34)
	II	59 (22)
	III	12 (4)
	IV	45 (16)
	V	63 (23)
Modified Fisher	0	5 (2)
	1	16 (6)
	2	14 (5)
	3	89 (33)
	4	146 (54)
Hydrocephalus		204 (76)
Rebleeding		10 (4)
Endovascular aneurysm repair, Coiling		125 (46)
Surgical aneurysm repair, Clipping		114 (42)
DCI		99 (37)
Angiographic vasospasm		107 (40)
Infection		129 (48)
mRS at discharge	0	16 (6)
	1	40 (15)
	2	38 (14)
	3	23 (9)
	4	30 (11)
	5	68 (25)
	6	55 (20)
SAPS II, median (LQ–UQ = IQR) (range)		31 (26–38 = 12) (9–65)
Mechanical ventilation		90 (33)
Tracheotomy		24 (9)
Catecholamine therapy		150 (56)
ARDS		3 (1)
Pulmonary edema		8 (3)
Takotsubo cardiomyopathy		15 (6)
ICU length of stay [d], median (LQ–UQ = IQR) (range)		13 (8–21 = 13) (0–68)
In-hospital mortality		55 (20)

Interquartile Range (IQR), Lower Quartile (LQ), Upper Quartile (UQ), modified Rankin Scale (mRS), World Federation of Neurological Surgeons SAH grading scale (WFNS), Simplified Acute Physiology Score II (SAPS II) at ICU admission, Acute Respiratory Distress Syndrome (ARDS).

**Table 4:** Infectious events in study cohort and causing effect on sepsis when using alternative sepsis criteria.

Infectious Event (N = 197)	N (% of Infectious Events)	Sepsis-Causing (N = 56); N (%)	Identified Pathogens if Sepsis-Causing ^1^ (N)
Urinary Tract Infection	73 (37)	7 (13)	*Escherichia coli* (4) *Proteus mirabilis* (2) *Enterobacter* spp. (2)
Pneumonia	49 (25)	32 (57)	*Staphylococcus aureus* (MSSA) (9) *Haemophilus influenzae* (6) *Klebsiella pneumonia* (4) *Enterobacter cloacae* (3) *Escherichia coli* (2) *Proteus mirabilis* (2) *Serratia marcescens* (2) *Citrobacter* spp. (2) *Pseudomonas aeruginosa* (1) *Streptococcus pneumoniae* (1) *Morganella morganii* (1) *Moraxella catarrhalis* (1) No pathogen identified in BAL (10)
Meningitis	27 (14)	4 (7)	*Klebsiella pneumonia* (2) *Escherichia coli* (1) *Proteus mirabilis* (1) *Serratia marcescens* (1)
Bloodstream Infection	21 (11)	9 (16)	*Staphylococcus aureus* (MSSA) (3) *Coagulase-negative Staphylococci* (3) *Klebsiella pneumonia* (1) *Escherichia coli* (1) *Proteus mirabilis* (1)
Gastrointestinal Infection	12 (6)	3 (5)	*Enterobacter cloacae* (1)
Central Line-Associated Infection	6 (3)	0	
Tracheobronchitis	5 (3)	1 (2)	n/a
Skin Infection	4 (2)	0	

Bronchoalveolar Lavage (BAL), not applicable (n/a) ^1^ A sepsis-causing infection could be associated with more than one pathogen.

**Table 5:** Frequency of sepsis and septic shock according to different criteria, associated bacteremia, and death.

Sepsis Criteria	Frequency of Sepsis in Overall Cohort (N = 270) N (%)	Frequency of Sepsis in Infected Patients (N = 129) N (%)	Bacteremia in Septic Patients N/Septic (%)	Deceased and Septic N/Septic (%)	Deceased and Non-Septic N/Non-Septic (%)
Sepsis-1					
sepsis	125 (46)	125 (97)	37/125 (30)	20/125 (16)	35/145 (24)
septic shock	105 (39)	105 (81)	32/105 (30)	20/105 (19)	35/165 (21)
Sepsis-3 (original)					
sepsis	60 (22)	60 (47)	21/60 (35)	11/60 (18)	44/210 (21)
septic shock	23 (9)	23 (18)	8/23 (35)	7/23 (30)	48/247 (19)
Sepsis-3 (modified)					
sepsis	60 (22)	60 (47)	25/60 (42)	13/60 (22)	42/210 (20)
septic shock	26 (10)	26 (20)	10/26 (38)	9/26 (34)	46/244 (19)
Alternative criteria					
sepsis	56 (21)	56 (43)	26/56 (46)	14/56 (25)	41/214 (19)
septic shock	28 (10)	28 (22)	11/28 (39)	10/28 (36)	45/242 (19)

## Data Availability

The data presented in this study are available on request from the corresponding author. The data are not publicly available due to restrictions of the data privacy office and ethics board’s decisions.

## Supplementary Materials

The following supporting information can be downloaded at: https://www.mdpi.com/article/10.3390/jcm11133873/s1, Table S1: Reasons for exclusion of patients during neuroradiological validation. Table S2: Organ dysfunctions associated with sepsis according to alternative sepsis criteria and relation to death. Table S3: Univariate and multivariate logistic regression models including different criteria for sepsis added separately to a model of SAH variables to investigate association with functional outcome (mRS) at discharge. Figure S1: Heatmap displaying Spearman’s rank correlation for variables that were significantly associated with the primary outcome (mRS) in logistic regression analysis. Table S4: Results of multicollinearity analysis of variables that were significantly associated with the primary outcome (mRS) in logistic regression analysis. Table S5: Sensitivity analysis excluding patients who deceased within 48 h after ICU admission: univariate and multivariate logistic regression models including different criteria for sepsis added separately to a model of SAH variables to investigate association with functional outcome (mRS) at discharge. Table S6: Univariate and multivariate logistic regression models including different criteria for sepsis added separately to a model of SAH variables to investigate association with in-hospital mortality. Table S7: Univariate and multivariate logistic regression models including different criteria for septic shock added separately to a model of SAH variables to investigate association with in-hospital mortality. Table S8: Sensitivity analysis excluding patients who deceased within 48 h after ICU admission: univariate and multivariate logistic regression models including different criteria for sepsis added separately to a model of SAH variables to investigate association with in-hospital mortality. Table S9: Sensitivity analysis excluding patients who deceased within 48 h after ICU admission: univariate and multivariate logistic regression models including different criteria for septic shock added separately to a model of SAH variables to investigate association with in-hospital mortality.
