# Supplementary material for: Comparative Analyses of the Impact of Different Criteria for Sepsis Diagnosis on Outcome in Patients with Spontaneous Subarachnoid Hemorrhage
Source: J Clin Med. 2022 Jul 4;11(13):3873. doi: 10.3390/jcm11133873 (PMC9267349; doi:10.3390/jcm11133873)
Supplement: Supplementary file 1 [file jcm-11-03873-s001.zip › jcm-1772628-supplementary.pdf]

Supplementary Materials

Comparative Analyses of the Impact of Different Criteria for Sepsis Diagnosis on Outcome in Patients with Spontaneous Subarachnoid Hemorrhage

Franz-Simon Centner, Mariella Eliana Oster, Franz-Joseph Dally, Johannes Sauter-Servaes, Tanja Pelzer, Jochen Johannes Schoettler, Bianka Hahn, Anna-Meagan Fairley, Amr Abdulazim, Katharina Antonia Margarete Hackenberg, Christoph Groden, Nima Etminan, Joerg Krebs, Manfred Thiel, Holger Wenz and Máté Elod Maros

Table S1. Reasons for exclusion of patients during neuroradiological validation [a].

**Reason for exclusion**	**N**
Traumatic SAH	14
Readmission: initial SAH ictus not in study period	9
No CT scan of initial SAH available	6
Transfer: SAH initially treated elsewhere	6
Ischemic stroke with SAH component	5
Intracerebral bleeding with SAH component	5
No SAH	3
SAH already consolidated in initial CT	2
SAH associated with cerebral neoplasia	2
Arterio-venous malformation with SAH component	2
SAH as surgical complication	2
Subdural hematoma with SAH component	1
SAH assocated with severe coagulation abnormalities due to	
ECMO therapy	5
cardiac resuscitation	3
sepsis	3
cerebritis	1
Missing data	1
Total	**70**

[a] reducing the number of study patients from 340 preselected by ICD-Code to 270

Table S2. Organ dysfunctions associated with sepsis according to alternative sepsis criteria and relation to death.

Organ system	Organ dysfunction<br>N (% of septic patients)	Deceased<br>N (% of patients affected by this organ dysfunction)
Cardiovascular	54 (96)	14 (26)
Respiration	38 (68)	12 (32)
Central nervous system	21 (38)	3 (14)
Renal	19 (34)	7 (37)
Gastrointestinal	13 (23)	4 (31)
Coagulation	8 (14)	5 (63)
Liver	6 (11)	4 (66)
Total	56 (100)	14 (25)

Table S3. Univariate and multivariate logistic regression models including different criteria for sepsis added separately to a model of SAH variables to investigate association with functional outcome (mRS) at discharge.

**Variable**	**Unfavorable outcome mRS 4-6 N=153 N (%)**	**Favorable outcome mRS 0-3 N=117 N (%)**	**Univariate p value**	**Multivariate model 1**[a]		**Multivariate model septic shock Sepsis-3_mod**		**Multivariate model alternative criteria for septic shock**	
				**adjusted OR (95% CI)**	**adjusted p value**	**adjusted OR (95% CI)**	**adjusted p value**	**adjusted OR (95% CI)**	**adjusted p value**
Age, mean (CLM)	61 (59-63)	54 (52-56)	**<.001**	1.04 (1.01-1.07)	**0.011**	1.04 (1.01-1.07)	**0.010**	1.04 (1.01-1.07)	**0.011**
Female	107 (70)	75 (64)	0.312						
Infection	91 (60)	38 (33)	**<.001**						
Pneumonia	41 (27)	5 (4)	**<.001**	7.62 (2.43-23.92)	**0.001**	6.2 (2.02-18.97)	**0.001**	4.7 (1.55-14.29)	**0.006**
WFNS 4-5	83 (54)	25 (21)	**<.001**	2.52 (1.29-4.9)	**0.007**	2.55 (1.32-4.94)	**0.005**	2.5 (1.3-4.79)	**0.006**
Mod. Fisher 3-4	146 (95)	89 (76)	**<.001**	4.18 (1.35-12.93)	**0.013**	4.12 (1.33-12.82)	**0.014**	3.34 (1.11-10.04)	**0.031**
Hydrocephalus	144 (94)	60 (51)	**<.001**	9.71 (4.02-23.44)	**<.001**	9.97 (4.12-24.16)	**<.001**	10.33 (4.27-24.98)	**<.001**
Rebleeding	9 (6)	1 (1)	0.062	7.67 (0.72-81.96)	0.092	7.16 (0.7-73.8)	0.098	6.35 (0.64-62.59)	0.113
Angiographic vasospasm	62 (41)	45 (39)	0.732						
DCI	64 (42)	35 (30)	**0.045**	1.84 (0.95-3.57)	0.070				
Updated DCI protocol	111 (73)	90 (77)	0.415	0.5 (0.24-1.05)	0.066	0.53 (0.25-1.12)	0.096		
Sepsis-1	89 (58)	36 (31)	**<.001**						
Sepsis-3_orig	47 (31)	13 (11)	**<.001**						
Sepsis-3_mod	47 (31)	13 (11)	**<.001**						
Alternative sepsis criteria	45 (29)	11 (9)	**<.001**						
Septic shock Sepsis-1	80 (52)	25 (21)	**<.001**						
Septic shock Sepsis-3_orig	18 (12)	5 (4)	**0.036**						
Septic shock Sepsis-3_mod	22 (14)	4 (3)	**0.005**			3.22 (0.92-11.35)	0.069		
Alternative criteria for septic shock	25 (16)	3 (3%)	**0.001**					5.04 (1.21-20.88)	**0.026**

Statistical significance highlighted in bold; *CLM* 95% Confidence Limit for the Mean, *DCI* delayed cerebral ischemia, *OR* Odds Ratio, *WFNS* World Federation of Neurological Surgeons SAH grading scale; [a] Model 1 shows all multivariable models where sepsis criteria did not reach significancy (sepsis-1, sepsis-3_orig, sepsis-3_mod, alternative sepsis, septic shock sepsis-1 and septic shock sepsis-3_orig).

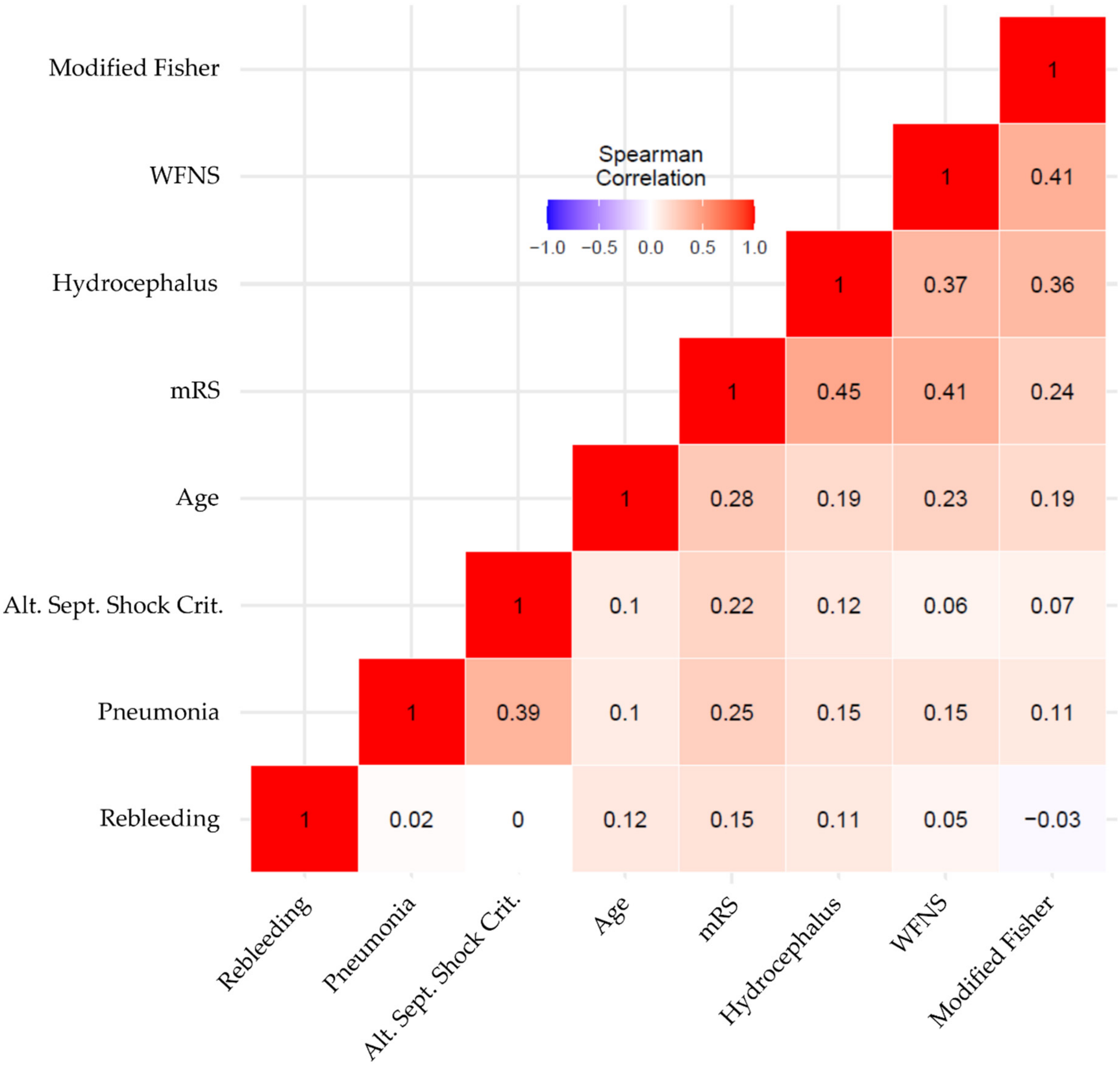

Figure S1. Heatmap displaying Spearman's rank correlation for variables that were significantly associated with the primary outcome (mRS) in logistic regression analysis. It is to note that mathematically it is not meaningful to code a factor variable (particularly binary factors e.g. rebleeding, pneumonia, alternative septic shock. criteria and hydrocephalus) as a numeric. Therefore, the results above should be interpreted with caution. *WFNS* World Federation of Neurological Surgeons SAH grading scale, *Alt. Sept. Shock Crit.* alternative criteria for septic shock.

Table S4. Results of multicollinearity analysis of variables that were significantly associated with the primary outcome (mRs) in logistic regression analysis [a].

Variable	Variance inflation factor (VIF)
Age	1.04
Pneumonia	1.09
WFNS	1.03
Modified Fisher	1.09
Hydrocephalus	1.02
Rebleeding	1.06
Alternative Septic Shock criteria	1.10

WFNS World Federation of Neurological Surgeons SAH grading scale;
[a] All variables had a variance inflation factor (VIF) < 1.15 indicating no relevant multicollinearity, which is usually defined as VIF > 5 (James, G.; Witten, D.; Hastie, T.; Tibshirani, R. An introduction to statistical learning. Springer: New York, USA, 2013; Vol. 112.). It is to note that mathematically VIF is not defined for categorical factor variables. However, collinearity between categorical data is more complex and less well understood than collinearity between numerical regressors. Therefore, the results above should be interpreted with caution as we treated the factors as numeric variables.

Table S5. Sensitivity analysis excluding patients who deceased within 48 hours after ICU admission*: Univariate and multivariate logistic regression models including different criteria for sepsis added separately to a model of SAH variables to investigate association with functional outcome (mRS) at discharge.

Variable	Unfavorable Outcome (mRS = 4-6) N=153; N (%)	Favorable Outcome (mRS = 0-3) N=117; N (%)	Univariate p value	Multivariate model 2[a]		Multivariate model septic shock Sepsis-3_mod		Multivariate model alternative criteria for septic shock	
				adjusted OR (95% CI)	adjusted p value	adjusted OR (95% CI)	adjusted p value	adjusted OR (95% CI)	adjusted p value
Age, mean (CLM)	60 (58-62)	54 (52-56)	**<.001**	1.03 (1-1.06)	**0.028**	1.04 (1.01-1.07)	**0.022**	1.04 (1.01-1.07)	**0.020**
Female	95 (71)	75 (64)	0.252						
Infection	90 (67)	38 (33)	**<.001**						
Pneumonia	40 (30)	5 (4)	**<.001**	7.63 (2.44-23.81)	**0.001**	5.71 (1.86-17.58)	**0.002**	4.96 (1.59-15.47)	**0.006**
WFNS 4-5	68 (51)	25 (21)	**<.001**	1.81 (0.92-3.57)	0.085	1.93 (0.98-3.81)	0.057	1.97 (1-3.89)	**0.049**
Modified Fisher 3-4	128 (96)	89 (76)	**<.001**	2.95 (0.99-8.79)	0.052	2.91 (0.96-8.8)	0.058	2.97 (0.97-9.04)	0.056
Hydrocephalus	127 (95)	60 (51)	**<.001**	13.46 (5.03-35.99)	**<.001**	13.3 (4.9-36.13)	**<.001**	13.47 (4.93-36.78)	**<.001**
Rebleeding	5 (4)	1 (1)	0.173						
Angiographic vasospasm	62 (46)	45 (39)	0.213						
DCI	64 (48)	35 (30)	**0.004**	2.18 (1.13-4.22)	**0.021**	2.03 (1.04-3.96)	**0.038**	1.98 (1.01-3.88)	**0.046**
Updated DCI protocol	97 (72)	90 (77)	0.411						
Sepsis-1	88 (66)	36 (31)	**<.001**						
Sepsis-3_orig	46 (34)	13 (11)	**<.001**						
Sepsis-3_rational	46 (34)	13 (11)	**<.001**						
Alternative sepsis criteria	44 (33)	11 (9)	**<.001**						
Septic shock Sepsis-1	79 (59)	25 (21)	**<.001**						
Septic shock Sepsis-3_orig	17 (13)	5 (4)	**0.025**						
Septic shock Sepsis-3_mod	21 (16)	4 (3)	**0.003**			3.01 (0.84-10.8)	0.090		
Alternative criteria for septic shock	24 (18)	3 (3)	**0.001**					4.37 (1-19.05)	**0.049**

Statistical significance highlighted in bold; *thereby comparable to Gonçalves B, Kurtz P, Turon R, Santos T, Prazeres M, Righy C, et al. Incidence and impact of sepsis on long-term outcomes after subarachnoid hemorrhage: a prospective observational study. Annals of Intensive Care. 2019;9(1):94.

CLM 95% Confidence Limit for the Mean, *DCI* delayed cerebral ischemia, *OR* Odds Ratio, *WFNS* World Federation of Neurological Surgeons SAH grading scale; [a] Model 2 shows all multivariable models where sepsis criteria did not reach significancy (Sepsis-1, Sepsis-3_orig, Sepsis-3_mod, alternative sepsis criteria, septic shock Sepsis-1 and septic shock Sepsis-3_orig).

Table S6. Univariate and multivariate logistic regression models including different criteria for sepsis added separately to a model of SAH variables to investigate association with in-hospital mortality.

Variable	Deceased N=55, N (%)	Survived N=215, N (%)	Univariate p value	Multivariate model 3[a]		Multivariate model alternative sepsis criteria	
				adjusted OR (95% CI)	adjusted p value	adjusted OR (95% CI)	adjusted p value
Age, mean (CLM)	63 (59-66)	57 (55-58)	**0.002**			1.02 (1-1.05)	0.100
Female	36 (66)	146 (67.9%)	0.729				
Infection	20 (36)	109 (50.7%)	0.060	0.26 (0.11-0.65)	**0.004**	0.19 (0.07-0.52)	**0.001**
Pneumonia	12 (22)	34 (15.8%)	0.292	2.95 (0.99-8.82)	0.052		
WFNS 4-5	38 (69)	70 (32.6%)	**<.001**	4.49 (2.2-9.18)	**<.001**	4.53 (2.19-9.37)	**<.001**
Modified Fisher 3-4	53 (96)	182 (84.7%)	**0.035**	5.82 (1.15-29.56)	**0.034**	4.74 (0.9-24.86)	0.066
Hydrocephalus	50 (91)	154 (71.6%)	**0.005**				
Rebleeding	6 (11)	4 (1.9%)	**0.005**	9.69 (2.07-45.25)	**0.004**	8.83 (1.81-43.05)	**0.007**
Angiographic vasospasm	15 (27)	92 (42.8%)	**0.038**				
DCI	16 (29)	83 (38.6%)	0.194				
Updated DCI protocol	33 (60)	168 (78.1%)	**0.007**	0.26 (0.13-0.55)	**<.001**	0.28 (0.14-0.59)	**0.001**
Sepsis-1	20 (36)	105 (48.8%)	0.100				
Sepsis-3_orig	11 (20)	49 (22.8%)	0.657				
Sepsis-3_mod	13 (24)	47 (21.9%)	0.778				
Alternative sepsis criteria	14 (26)	42 (19.5%)	0.335			4.24 (1.35-13.31)	**0.013**

Statistical significance highlighted in bold

CLM 95% Confidence Limit for the Mean, *DCI* delayed cerebral ischemia, *OR* Odds Ratio, *WFNS* World Federation of Neurological Surgeons SAH grading scale

[a] Model 3 shows all multivariable models where sepsis criteria did not reach significancy (Sepsis-1, Sepsis-3_orig, Sepsis-3_mod).

Table S7. Univariate and multivariate logistic regression models including different criteria for septic shock added separately to a model of SAH variables to investigate association with in-hospital mortality.

**Variable**	**Deceased N=55 N (%)**	**Survived N=215 N (%)**	**Uni-variate p value**	**Multivariate model septic shock Sepsis-1**		**Multivariate model septic shock Sepsis-3_orig**		**Multivariate model septic shock Sepsis-3_mod**		**Multivariate model alternative criteria for septic shock**	
				**adjusted OR (95%CI)**	**adjusted p value**	**adjusted OR (95%CI)**	**adjusted p value**	**adjusted OR (95%CI)**	**adjusted p value**	**adjusted OR (95%CI)**	**adjusted p value**
Age, mean (CLM)	63 (59-66)	57 (55-58)	**0.002**			1.03 (1-1.06)	0.079	1.03 (1-1.06)	0.070		
Female	36 (66)	146 (68)	0.729								
Infection	20 (36)	109 (51)	0.060	0.26 (0.11-0.65)	**0.004**	0.29 (0.13-0.64)	**0.002**	0.23 (0.1-0.53)	**0.001**	0.23 (0.1-0.53)	**0.001**
Pneumonia	12 (22)	34 (16)	0.292	2.95 (0.99-8.82)	0.052						
WFNS 4-5	38 (69)	70 (33)	**<.001**	4.49 (2.2-9.18)	**<.001**	4.53 (2.19-9.38)	**<.001**	5.09 (2.41-10.73)	**<.001**	5.54 (2.66-11.54)	**<.001**
Modified Fisher 3-4	53 (96)	182 (85)	**0.035**	5.82 (1.15-29.56)	**0.034**	5.19 (0.99-27.2)	0.051	5.23 (0.97-28.11)	0.054	6.11 (1.16-32.08)	**0.033**
Hydro-cephalus	50 (91)	154 (72)	**0.005**								
Rebleeding	6 (11)	4 (2)	**0.005**	9.69 (2.07-45.25)	**0.004**	8.95 (1.85-43.3)	**0.006**	9.86 (1.96-49.52)	**0.005**	11.45 (2.34-56)	**0.003**
Angiographic vasospasm	15 (27)	92 (43)	**0.038**								
DCI	16 (29)	83 (39)	0.194								
Updated DCI protocol	33 (60)	168 (78)	**0.007**	0.26 (0.13-0.55)	**<.001**	0.28 (0.13-0.58)	**0.001**	0.26 (0.12-0.56)	**0.001**	0.27 (0.13-0.57)	**<.001**
Septic shock Sepsis-1	20 (36)	85 (40)	0.667								
Septic shock Sepsis-3_orig	7 (13)	16 (7)	0.216			4.22 (1.25-14.25)	**0.020**				
Septic shock Sepsis-3_mod	9 (16)	17 (8)	0.063					7.58 (2.33-24.67)	**0.001**		
Alternative criteria for septic shock	10 (18)	18 (8)	**0.038**							7.48 (2.35-23.74)	**<.001**

Statistical significance highlighted in bold
CLM 95% Confidence Limit for the Mean, *DCI* delayed cerebral ischemia, *OR* Odds Ratio, *WFNS* World Federation of Neurological Surgeons SAH grading scale.

Table S8. Sensitivity analysis excluding patients who deceased within 48 hours after ICU admission: Univariate and multivariate logistic regression models including different criteria for sepsis added separately to a model of SAH variables to investigate association with in-hospital mortality.

**Variable**	**Deceased N=55, N (%)**	**Survived N=215, N (%)**	**Univariate p value**	**Multivariate model 4[a]**		**Multivariate model alternative sepsis criteria**	
				**adjusted OR (95% CI)**	**adjusted p value**	**adjusted OR (95% CI)**	**adjusted p value**
Age, mean (CLM)	60 (55-65)	57 (55-58)	0.142				
Female	24 (67)	146 (68)	0.883				
Infection	19 (53)	109 (51)	0.817				
Pneumonia	11 (31)	34 (16)	**0.037**	2.11 (0.87-5.11)	0.097		
WFNS 4-5	23 (64)	70 (33)	**0.001**	3.09 (1.41-6.77)	**0.005**	3.41 (1.57-7.43)	**0.002**
Modified Fisher 3-4	35 (97)	182 (85)	0.073	6.32 (0.77-51.65)	0.086	5.86 (0.72-47.99)	0.099
Hydrocephalus	33 (92)	154 (72)	**0.018**				
Rebleeding	2 (6)	4 (2)	0.201				
Angiographic vasospasm	15 (42)	92 (43)	0.900				
DCI	16 (44)	83 (39)	0.508				
Updated DCI protocol	19 (53)	168 (78)	**0.002**	0.23 (0.1-0.51)	**<.001**	0.25 (0.11-0.54)	**0.001**
Sepsis-1	19 (53)	105 (49)	0.662				
Sepsis-3_orig	10 (28)	49 (23)	0.515				
Sepsis-3_mod	12 (33)	47 (22)	0.137				
Alternative sepsis criteria	13 (36)	42 (20)	**0.029**			2.13 (0.94-4.82)	0.069

Statistical significance highlighted in bold

CLM 95% Confidence Limit for the Mean, *DCI* delayed cerebral ischemia, *OR* Odds Ratio, *WFNS* World Federation of Neurological Surgeons SAH grading scale

[a] Model 4 shows all multivariable models where sepsis criteria did not reach significancy (Sepsis-1, Sepsis-3_orig, Sepsis-3_mod).

Table S9. Sensitivity analysis excluding patients who deceased within 48 hours after ICU admission: Univariate and multivariate logistic regression models including different criteria for septic shock added separately to a model of SAH variables to investigate association with in-hospital mortality.

Variable	Deceased N=55 N (%)	Survived N=215 N (%)	Uni-variate p value	Multivariate model septic shock Sepsis-1		Multivariate model septic shock Sepsis-3_orig		Multivariate model septic shock Sepsis-3_mod		Multivariate model alternative criteria for septic shock	
				**adjusted OR (95%CI)**	**adjusted p value**	**adjusted OR (95%CI)**	**adjusted p value**	**adjusted OR (95%CI)**	**adjusted p value**	**adjusted OR (95%CI)**	**adjusted p value**
Age, mean (CLM)	60 (55-65)	57 (55-58)	0.142								
Female	24 (67)	146 (68)	0.883								
Infection	19 (53)	109 (51)	0.817								
Pneumonia	11 (31)	34 (16)	**0.037**	2.11 (0.87-5.11)	0.097						
WFNS 4-5	23 (64)	70 (33)	**0.001**	3.09 (1.41-6.77)	**0.005**	3.46 (1.59-7.52)	**0.002**	3.82 (1.71-8.5)	**0.001**	3.79 (1.7-8.44)	**0.001**
Modified Fisher 3-4	35 (97)	182 (85)	0.073	6.32 (0.77-51.65)	0.086	6.7 (0.81-55.61)	0.078	6.79 (0.78-58.89)	0.082	6.65 (0.77-57.7)	0.086
Hydro-cephalus	33 (92)	154 (72)	**0.018**								
Rebleeding	2 (6)	4 (2)	0.201					5.73 (0.71-46.27)	0.102	5.83 (0.73-46.87)	0.097
Angiographic vasospasm	15 (42)	92 (43)	0.900								
DCI	16 (44)	83 (39)	0.508								
Updated DCI protocol	19 (53)	168 (78)	**0.0018**	0.23 (0.1-0.51)	**<.001**	0.24 (0.11-0.54)	**0.001**	0.23 (0.1-0.52)	**<.001**	0.24 (0.11-0.53)	**0.001**
Septic shock Sepsis-1	19 (53)	85 (40)	0.138								
Septic shock Sepsis-3_orig	6 (17)	16 (7)	0.078			2.55 (0.85-7.64)	0.095				
Septic shock Sepsis-3_mod	8 (22)	17 (8)	**0.011**					4.12 (1.49-11.38)	**0.006**		
Alternative criteria for septic shock	9 (25)	18 (8)	**0.005**							4.28 (1.6-11.5)	**0.004**

Statistical significance highlighted in bold
CLM 95% Confidence Limit for the Mean, *DCI* delayed cerebral ischemia, *OR* Odds Ratio, *WFNS* World Federation of Neurological Surgeons SAH grading scale.
